# Supplementary material for: Do non-pharmaceutical policies in response to COVID-19 affect stock performance? Evidence from Malaysia stock market return and volatility
Source: PLoS One. 2023 Jan 31;18(1):e0277252. doi: 10.1371/journal.pone.0277252 (PMC9888690; doi:10.1371/journal.pone.0277252)
Supplement: Attachment — Submitted filename: Response to reviewers.docx [file pone.0277252.s001.docx]

**Rebuttal Letter**

We thank the editor and the two viewers for their comments on our manuscript. Below is our response to each point raised by the academic editor and reviewers. We hope that we satisfyingly addressed them and that the manuscript will be now suited for publication.

Sincerely,

On behalf of all authors,

Racquel Rowland

**Academic editor:**

1. **Please ensure that your manuscript meets PLOS ONE's style requirements, including those for file naming.**

The manuscript has been altered to adhere to PLOS ONE style requirement.

2. **PLOS ONE does not copy edit accepted manuscripts.**

The manuscript was presented and written in Standard English.

3. **In your Data Availability statement, you have not specified where the minimal data set underlying the results described in your manuscript can be found. PLOS defines a study's minimal data set as the underlying data used to reach the conclusions drawn in the manuscript and any additional data required to replicate the reported study findings in their entirety. All PLOS journals require that the minimal data set be made fully available. For more information about our data policy, please see** [**http://journals.plos.org/plosone/s/data-availability**](http://journals.plos.org/plosone/s/data-availability)**.**

**Upon re-submitting your revised manuscript, please upload your study’s minimal underlying data set as either Supporting Information files or to a stable, public repository and include the relevant URLs, DOIs, or accession numbers within your revised cover letter. For a list of acceptable repositories, please see** [**http://journals.plos.org/plosone/s/data-availability#loc-recommended-repositories**](http://journals.plos.org/plosone/s/data-availability#loc-recommended-repositories)**. Any potentially identifying patient information must be fully anonymized.**

**Important: If there are ethical or legal restrictions to sharing your data publicly, please explain these restrictions in detail. Please see our guidelines for more information on what we consider unacceptable restrictions to publicly sharing data:** [**http://journals.plos.org/plosone/s/data-availability#loc-unacceptable-data-access-restrictions**](http://journals.plos.org/plosone/s/data-availability#loc-unacceptable-data-access-restrictions)**. Note that it is not acceptable for the authors to be the sole named individuals responsible for ensuring data access.**

**We will update your Data Availability statement to reflect the information you provide in your cover letter.**

**Additional Editor Comments:**

**I recommend the authors to read carefully the reviewers' comments. In addition, I would like to suggest to better explain their contribution to the empirical literature. There are several papers analyzing the effect of Covid-19 and mitigation policies on stock markets, bond markets, cryptocurrencies, etc. Therefore, authors could summarize some papers, explaining findings and trying to find some common pattern with previous research.**

The empirical finding from previous papers has been updated in the empirical literature and can be seen on page 5, lines 99-135.

**I think the definition of volatility is not correct. The authors define it as: log|Ret|_t. However, if RET=0.5, then log|RET| will be negative. This could be solved just by redefining RET=log(P_t-Pt-1), and then VOL=|RET|. Alternatively, you could define it as |log|RET||.**

The measurement of volatility was re-adjusted and re-calculated based on the correct definition suggested by the editor and can be seen on page 5, line 186.

**Finally, and in line with Reviewer 1, I suggest to update data and results.**

The data has now updated until 31^st^ May 2022 at page 1, line 17 and page 5, line 162 whereas new results have generated and referred to page 6-10, lines 221-393.

**Reviewer #1**

**The abstract starts with “Before vaccination policy introduced in the early 2021, Malaysian government has made the decision to impose non-pharmaceutical intervention to contain the deadly virus.” I believe there is no need for it as all the countries did the same. Please focus on the aim, data, methodology and findings in the abstract.**

The abstract starts with ‘Before vaccination policy introduced in the early 2021, Malaysian government has made the decision to impose non-pharmaceutical intervention to contain the deadly virus…” was removed and amended with the necessary information as requested by reviewer which can refer to page 1, line 15-16.

**This topic has been widely studied both in single country context and in multi-country settings. So what is new in the paper? The paper lacks a contribution. Even if the authors believe that there is a contribution, it is not written in the introduction. How this paper differs from the previous studies? Moreover why Malaysia? Why it is interesting to see single country findings while we have studies using multi-country data? Simply, the paper lacks a contribution. Same topics have been studied before. It is a replication for Malaysia.**

The explanation on choosing Malaysia stock market as focus of the study is explained in the contribution of study which can be found on page 4 lines 136-151.

**In the introduction, authors say that: “With the inconsistent standard operating system going on in Malaysia, the accumulated number of COVID-19 confirmed cases is accelerated at exponential rate on a daily basis”. There is no exponential growth of cases now. Please pay attention to your arguments.**

Yes, there was no exponential growth of cases now. However, the sentence was incomplete as the exponential growth of cases was referring to the spike of covid-19 cases during the third wave of pandemic as the Malaysian government started to ease the restriction policies. It was reported that the third wave started in September 2020 which origins from two big clusters, namely the Benteng Lahad Datu cluster,in Sabah state and Kedah’s Tembok cluster. The sudden spike was due to poor compliance with COVID-19 SOPs. Hence, this statement has amended on page 4, line 143-153.

**The starting period of the data is clarified but the ending of the data period is not clear. Why the data ends on 30th November, 2021? I am not asking for a data from May 2022 but it is now 6 months old and if and when it is published, it will be 8 months old (at least).**

The ending period of the data now has updated to the latest which is until May 31, 2022 as requested by the reviewers and can refer to page 1, line 17 and page 5, line 162. The reason for choosing this date as ending period because first, some of the macroeconomic variables data availability such as interest rate, unemployment rate, industrial production index and consumer price index are only available until 31^st^ May, 2022 and secondly, it is also for synchronization purpose.

**The use of the interaction of growth in Covid-19 case and SI is not clear to me.**

The moderation effect generated from the interaction variables will be able to explain how the stringency index which proxied for social distancing measure enable to moderate the effect of covid-19 growth case on the stock return and volatility. As the government impose stringent social distancing measure such as lockdown and restriction movement were introduced to combat the disease, it is believed that such policy will reduce the infection rate of covid-19. Following the policy implementation, the investor is able to restructure their portfolio accordingly. The explanation on the use of interaction variable of COVID-19 growth rate and stringency index is amended and can be found on page 6, lines 204-208.

**There is a problem with the use of notations, subscripts.**

The amendment is made to the equation on page 5, lines 184 and page 6, line 201, 213 with the correct use of notation and subscripts.

**Do you consider the autocorrelation?**

This study bootstraps the standard errors for the OLS coefficients, using the empirical residuals. As such, the resulting t-value and marginal significance value (p) are valid regardless of the nature of the residuals.

**The paper lacks robustness checks. You can use different measures of volatility. You can use growth of deaths instead of growth of cases.**

Good point. The daily COVID-19 death growth variable will give an additional information for market participants in repositioning their portfolio since the resumption of economic activity relies heavily on how well the government in handling the deadly disease by preventing the daily death growth from accelerating. Hence, the employment of daily covid-19 death growth is introduced to all three model specifications and its result can refer to page 20, Panel A in Table 10. The discussion of the empirical findings can be found on page 9, lines 355-362

**Do you control for the day of the week effect?**

The weekday dummies variable is introduced as additional variable to all three model specifications in order to control for day-of-the-week and the explanation of the effect can be found on page 10 lines 268-371 and the results can be found on page 21, Table 11.

**You can also check if different industries are differently affected.**

Good point. As the government imposed social distancing measure, many sectors have severely affected by the policies which disrupt the economic activity. Thus, the use of different industries or sectors was introduced as dependent variables to capture the diverse impact in response to the non-pharmaceutical intervention by government. Hence, the results and discussion for the policy implication on different sectors can refer to Table 4-10 on page 14-19 and page 9, lines 320-348.

**Vaccination should be considered as it becomes an important factor.**

Good point. The employment of vaccination variable into the model specification enables to identify which policies works best at mitigating the disease by comparing it with the non-pharmaceutical strategy proxy in determining the stock return as well as volatility. Hence, the log (daily vaccination) variable is introduced as additional variable to all three model specifications with alternative dependent measure. The results and discussion can be referred to page 9-10, lines 359-368 and page 20, Panel B in Table 10 respectively.
