# Supplementary material for: Do non-pharmaceutical policies in response to COVID-19 affect stock performance? Evidence from Malaysia stock market return and volatility
Source: PLoS One. 2023 Jan 31;18(1):e0277252. doi: 10.1371/journal.pone.0277252 (PMC9888690; doi:10.1371/journal.pone.0277252)
Supplement: Attachment — Submitted filename: Rebuttal Letter_Response to reviewer.docx [file pone.0277252.s002.docx]

**Rebuttal Letter**

We thank the editor and the reviewer for their comments on our manuscript. Below is our response to each point raised by the academic editor and reviewers. We hope that we satisfyingly addressed them and that the manuscript will be now suited for publication.

Sincerely,

On behalf of all authors,

Racquel Rowland

**Reviewer #1**

**To use “On the other hand”, you need to use on the one hand, please drop this from the abstract.**

The use “on the other hand” was removed and changed to “on the one hand’ in the abstract which can refer to line 21 on page 1.

**Please add the citations for the variables you used in the analysis.**

The citation for the additional variables in the model specification was amended and can refer to line 371 to 397 page 10.

**Robustness tests add value to the paper. It is important to see if the findings for Malaysia is different from other countries. In this sense, authors can cite some additional papers for comparison.**

The amendment can refer to line 371 to 397 page 10.

**Please check the citation and reference match, Rouatbi et al. (2021) is cited but not in the references.**

The reference for the citation was amended and can refer to line 45 at page 23.

**This is a misunderstanding of the robustness idea. For day of the week effect, you introduce those day dummies to show if your main findings remain the same or not, not to comment on day of week effect. And we see that CGRATE*SI becomes insignificant. So, your findings are not robust to alternative measures.**

The robustness checks on the social distancing measure indicators was amended in the analysis when weekday dummies was introduce to the model specification and can refer to line 393 to 397 at page 11.

**“Similarly, a one percent increase 356 in daily covid-19 death growth will trigger the volatility from 0.001% to 0.004% but not statistically significant.” If a coefficient is not significant, it has no effect so there is no need to mention triggering effect. Simply, there is no significant effect of covıd-10 death growth. “This can explain that daily covid-19 death growth is less significant in explaining the stock market returns and volatility in the context of Malaysia” I couldn’t understand this, death is not significant, it is not less significant. Please read the paper careful and use proper wordings. The paper needs a proofing, a real one.**

The sentence started with ““Similarly, a one percent increase 356 in daily covid-19 death growth will trigger the volatility from 0.001% to 0.004% but not statistically significant.” was removed from the paragraph as suggested by the reviewer.
